# Reconstruction with a double-constrained implant design after complex shoulder extra-articular resection

**DOI:** 10.1186/s12957-023-03173-9

**Published:** 2023-09-18

**Authors:** Jan Lesensky, Ana C. Belzarena, Matej Daniel

**Affiliations:** 1 Department of Orthopaedics, First Medical Faculty, University Hospital Na Bulovce, Charles University, Prague, Czech Republic; 2 Department of Orthopedic Surgery, University of Missouri, Columbia, USA; 3 Department of Mechanics, Biomechanics, and Mechatronics, Faculty of Mechanical Engineering, Czech Technical University in Prague, Prague, Czech Republic

**Keywords:** Shoulder reconstruction, Tumor, Sarcoma, Extra-articular resection

## Abstract

**Background:**

Approximately, one-third of patients with tumors of proximal humerus will require an extra-articular resection to achieve oncologic margins. This procedure yields poor functional outcomes with a considerable rate of revisions. Unconstrained implants are prone to instability hindering also function of the elbow and hand, whereas constrained shoulder reconstructions suffer from early aseptic loosening of the glenoid component due to bone overload. The purpose of this study was to develop a constrained implant suitable for extra-articular resection with loss of function in deltoid and rotator cuff, which would provide both stability and passive motion, whilst also decreasing the risk of aseptic loosening of the glenoid component.

**Methods:**

In cooperation with Czech Technical University in Prague, we devised an implant consisting of two constrained joints in series connected by a dumbbell piece. The biomechanical analysis showed a reduction of load transfer to the glenoid component with a torque of 8.6 Nm capable of generating an 865-N pulling force on bone screw to just 0.07 Nm, hence shielding the glenoid component from undesired forces and decreasing the risk of aseptic loosening. Three patients with extra-articular resection with a total loss of function of both rotator cuff and deltoid muscle received this type of reconstruction. The average follow-up was 16 months.

**Results:**

The surgical technique is straightforward. The surgery took 175 min on average with average blood loss of 516 ml. There were no surgical- or implant-related complications. All three patients were pain-free and had a stable shoulder joint after the reconstruction. All had fully functional elbow, wrist, and hand joints. The average Musculoskeletal Tumor Society (MSTS) score was 21/30 (70%). All patients were pleased with the results.

**Conclusion:**

The presented innovative implant design has demonstrated to be a promising alternative for reconstruction in these challenging cases.

## Introduction

The proximal humerus is a frequent location for bone tumors [1, 2]. These include primary bone sarcomas, commonly seen in children and young adults, as well as metastatic disease [3, 4]. For those patients, the treatment gold standard is tumor resection with adequate margins along with reconstruction to salvage the limb [5, 6]. Several alternatives exist nowadays for the reconstruction of proximal humerus, however, with unsatisfactory outcomes and considerable rates of revisions [7, 8]. Furthermore, approximately a third of the patients will require an extra-articular resection to achieve oncologic margins [9, 10]. This extensive procedure has been associated with poor functional outcomes and high complication rates [11]. The glenohumeral joint has the largest range of motion in the human body with six degrees of freedom [12, 13]. The stability of the shoulder joint depends mostly on dynamic stabilization mechanisms (rotator cuff, scapulohumeral rhythm) that allow movement with a large range of motion. The shoulder arthroplasty also relies on such mechanisms, e.g., success of the reverse shoulder arthroplasty depends on the deltoid and other remaining muscles to achieve movement and stability [14]. However, most of these mechanisms are rendered non-functional as they ought to be sacrificed for the sake of obtaining tumor-free margins [15]. This scenario makes all the known available reconstruction options available, such as endoprosthetic replacements, allografts, or allograft-prosthesis composites, prone to instability. Moreover, oftentimes, the deltoid muscle and/or axillary nerve is sacrificed during the tumor resection, creating a contraindication for many of the reconstructive options [16]. This scenario may leave the constrained shoulder arthroplasty as the sole plausible form of reconstruction [17], which is also prone to early aseptic loosening [8, 18] that limits its usefulness. Alternatively, a shoulder fusion using vascularized fibula can be pursued [19], which is a lengthy, complicated procedure with significant risks and yields a rigid shoulder, which further limits the utilization of the limb. Ideally, the patient should be allowed to position their hand freely in space whilst also having a stable shoulder support for function of the elbow and hand.

The purpose of this study was to develop a novel implant design that has the advantages of constrained shoulder joint whilst being less prone to aseptic loosening of the glenoid component. We present the novel implant, the surgical technique with pitfalls, and short-term results of three patients.

## Materials and methods

### Biomechanical design analysis

The implant design was developed in cooperation with the Department of Mechanics, Biomechanics and Mechatronics at the Czech Technical University in Prague. The design is based on the knowledge of biomechanics and failure mode of the current designs. In order to study the biomechanics of the shoulder joint replacement with a nonfunctional deltoid muscle and rotator cuff, we adopted a three-dimensional musculoskeletal model of the shoulder developed in OpenSim [20]. Muscle paths were adjusted to produce moment arms bounded by measurements from cadaver experiments [21].

**Fig. 1:**
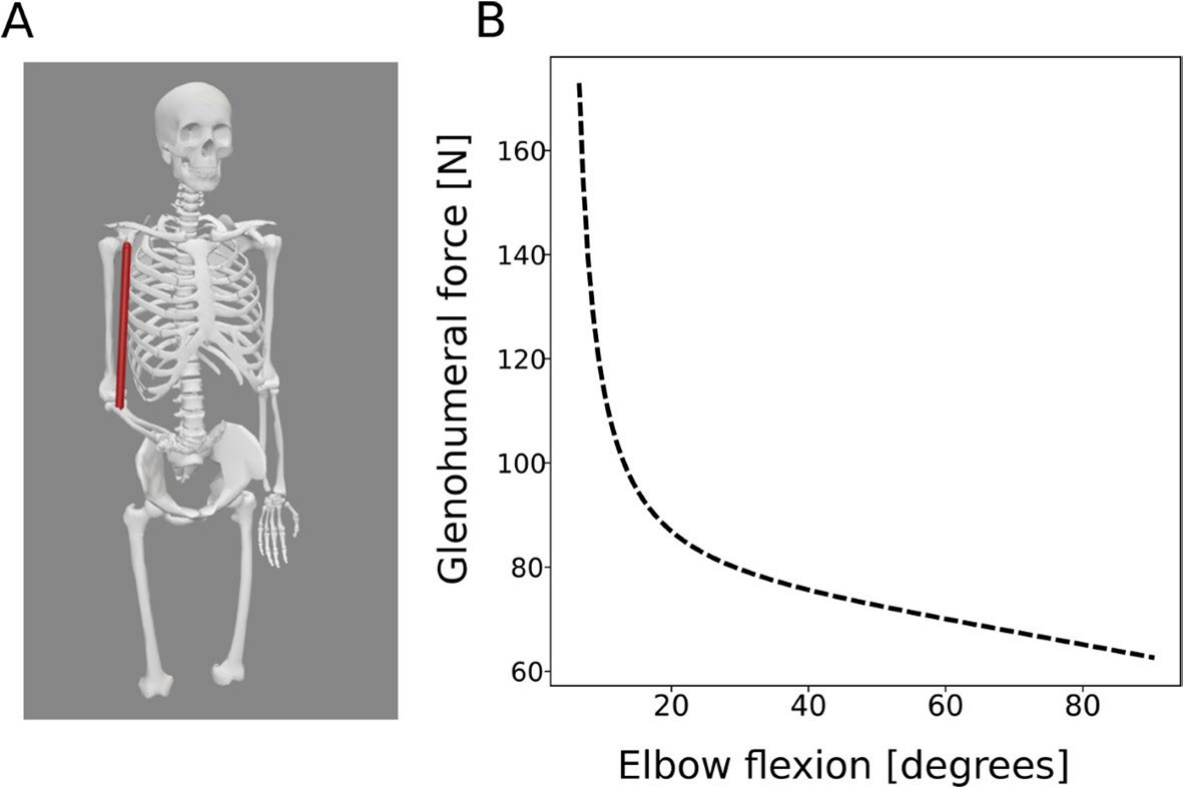
**A** Musculoskeletal model of the shoulder. **B** Glenohumeral loading force during elbow flexion

**Fig. 2:**
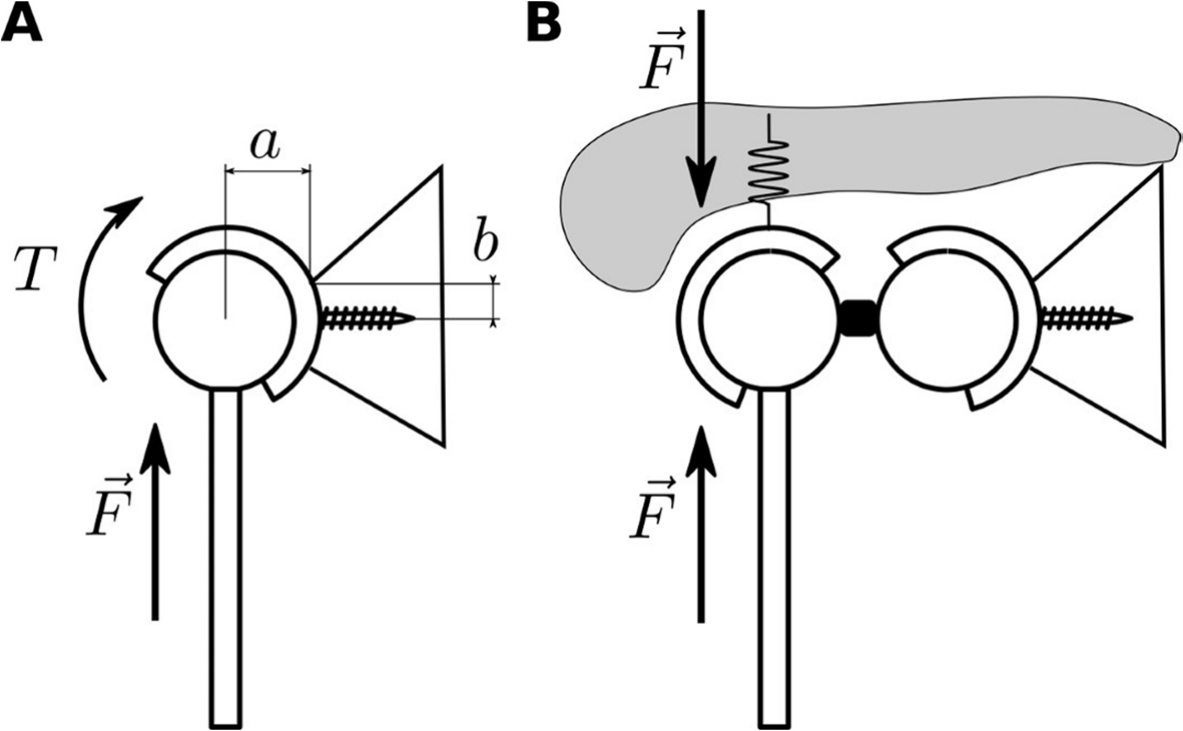
Scheme of the constrained shoulder joint replacement loading. **A** In single ball joint configuration, the force *F* is transmitted directly through the joint, induces torque *T* that pull off anchoring cortical screw. **B** In double bearing configuration, the loading force *F* is compensated by deformation of soft tissue, and torque loading of glenoidal component is considerably reduced

The primary implant loading is represented by the short head of biceps (Fig. 1A). A scenario was adopted, where the shoulder joint acts as passive joint and the biceps muscle contributes to elbow flexion. The upper arm is stabilized by contact with the torso, and no motion in the shoulder joint is assumed. Inverse dynamic analysis with static reduction was employed to analyze glenohumeral forces. In these settings, the primary glenohumeral loading induced by the short head of biceps is a vertical force ranging from 63 N to maximum force of 173 N in 90 to 7° of elbow flexion, respectively (Fig. 1B). The primary component of the muscular loading force is the vertical force accounting for 87%. In a constrained implant, the force is transmitted through the constrained humeral head directly into the glenoidal component. The critical part of the construction is the anchoring of the glenoid component to the scapula by either a single or a set of cortical screws. For the described loading, the glenoid component acts as a lever that pulls off the cortical screws from the bone. In a typical scenario, the glenoidal component is anchored by a cortical screw at a distance of “*b* = 1 cm” from the scapular bone edge. The center of rotation is located approx. “*a* = 5 cm” from the scapular bone edge (see, for example, Bayley-Walker prosthesis [22], Fig. 2A) that gives mechanical lever advantage of *a*/*b* = 5. Therefore, the torque as high as 8.6 Nm on glenoidal component is acting that generates 865-N pull-off force at the cortical screw. Repeated pull-off loading might result in screw loosening or its mechanical failure [23].

### Final implant design

From the above follows that it would be beneficial to decrease the acting torque to increase the lifetime of a constrained glenohumeral joint replacement and to substitute for the lacking soft tissues that act in way where the joint torque is transmitted through coordinated muscle action. Therefore, an alternative design was proposed that is based on a double bearing approach. The novel design allows vertical displacement of the joint to interact with the overlaying soft tissues (Fig. 2B). The loading force is transmitted not through the constrained joint but through the soft tissue that minimizes the torque loading of the glenoidal components. The torque transmitted through the joint was further minimized by decreasing the ball radius and using low friction DLC coating interacting with the polymer inlay made of PEEK. The torque loading of the glenoid component is attributed to the friction torque. For a transversal force of 22 N, a radius of ball of 1 cm, and a friction coefficient 0.3 [24], the torque is lower than 0.07 Nm.

**Fig. 3:**
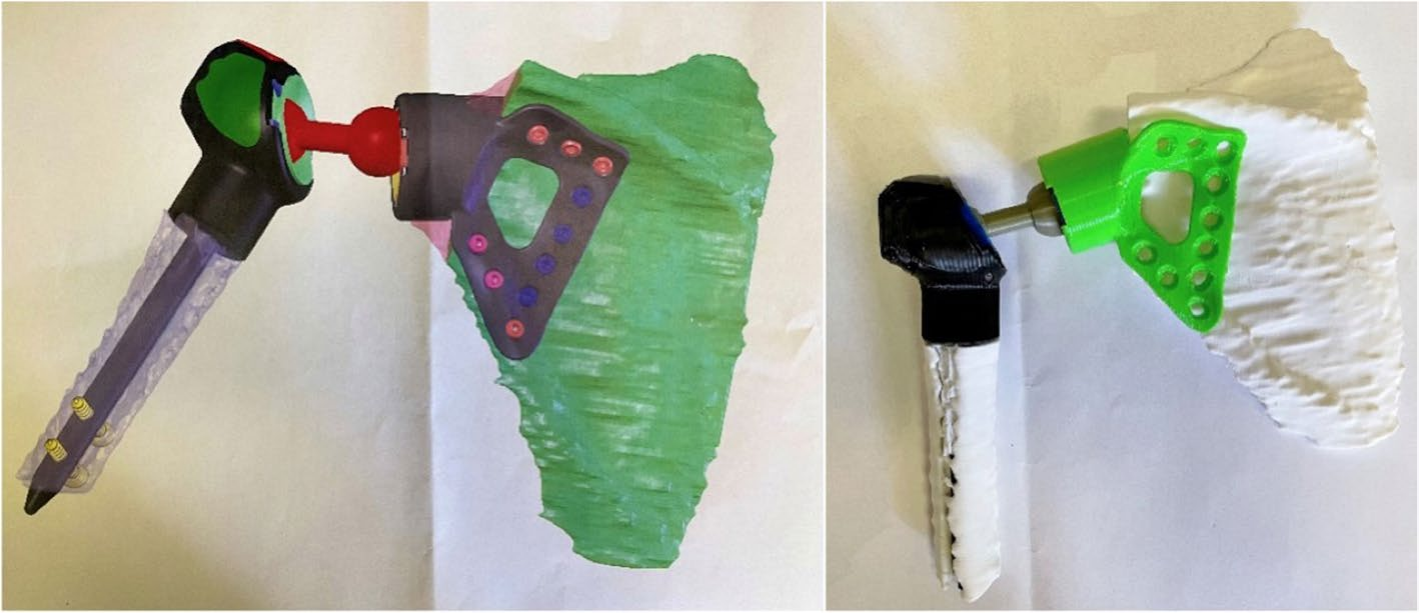
Preliminary 3D-printed dummy. Pictures demonstrating the initial design and printed dummy subject to team approval prior to definitive surgery. Dumbbell connector linking the humeral and scapular components

The final implant is composed of two spherical constrained joints in series with a “dumbbell” connector. The implant is additively manufactured from titanium alloy Ti6Al4V with a diamond-like carbon surface and is freely combinable with modular humeral components. The dumbbell connector is made of polished steel (Figs. 3 and 5).

### Patients

Patients who qualified for the indications of this novel implant with planned resection of the axillary nerve and rotator cuff tendons were presented with this reconstruction alternative as well as all other currently available reconstruction options along its risks and benefits. Three patients, 2 males and a female of average age of 44 years (17 to 72 years), agreed to participate in the study (Table 1). Patients were consented for surgery and received this custom-made shoulder prosthesis as part of their standard treatment protocol. There were no other patients receiving this prosthesis at our institution. Two patients had diagnosis of telangiectatic osteosarcoma with a concomitant pathological fracture, and one had a synovial sarcoma post-initial unplanned resection. All three patients required resection of the axillary nerve to achieve oncologic margins as well as an extra-articular resection including transection of rotator cuff tendons at the level of the scapular neck. All surgeries were performed by the same experienced surgical team. The average follow-up time was 26 months (22 to 32 months).

**Table 1:** Patient and tumor characteristics

Patient	Gender	Age	Diag	Path. fract	Surgical time	EBL	F/U	Pain	Complications	Oncol. outcome	MSTS score
Patient 1	Male	43	OST	Yes	185 min	1000 mL	32 mo	0/10	Implant protrusion	NED	21/30
Patient 2	Male	17	OST	Yes	190 min	400 mL	23 mo	0/10	Implant protrusion	NED	22/30
Patient 3	Female	72	SS	No	150 min	150 mL	22 mo	0/10	None	NED	20/30

*Diag* diagnosis, *OST* osteosarcoma, *SS* synovial sarcoma, *Path. fract* pathological fracture, *EBL* estimated blood loss, *F/U* follow-up time, *Oncol* outcomes oncologic outcomes, *NED* no evidence of disease, *MSTS score* Musculoskeletal Tumor Society score

### Surgical technique

**Fig. 4:**
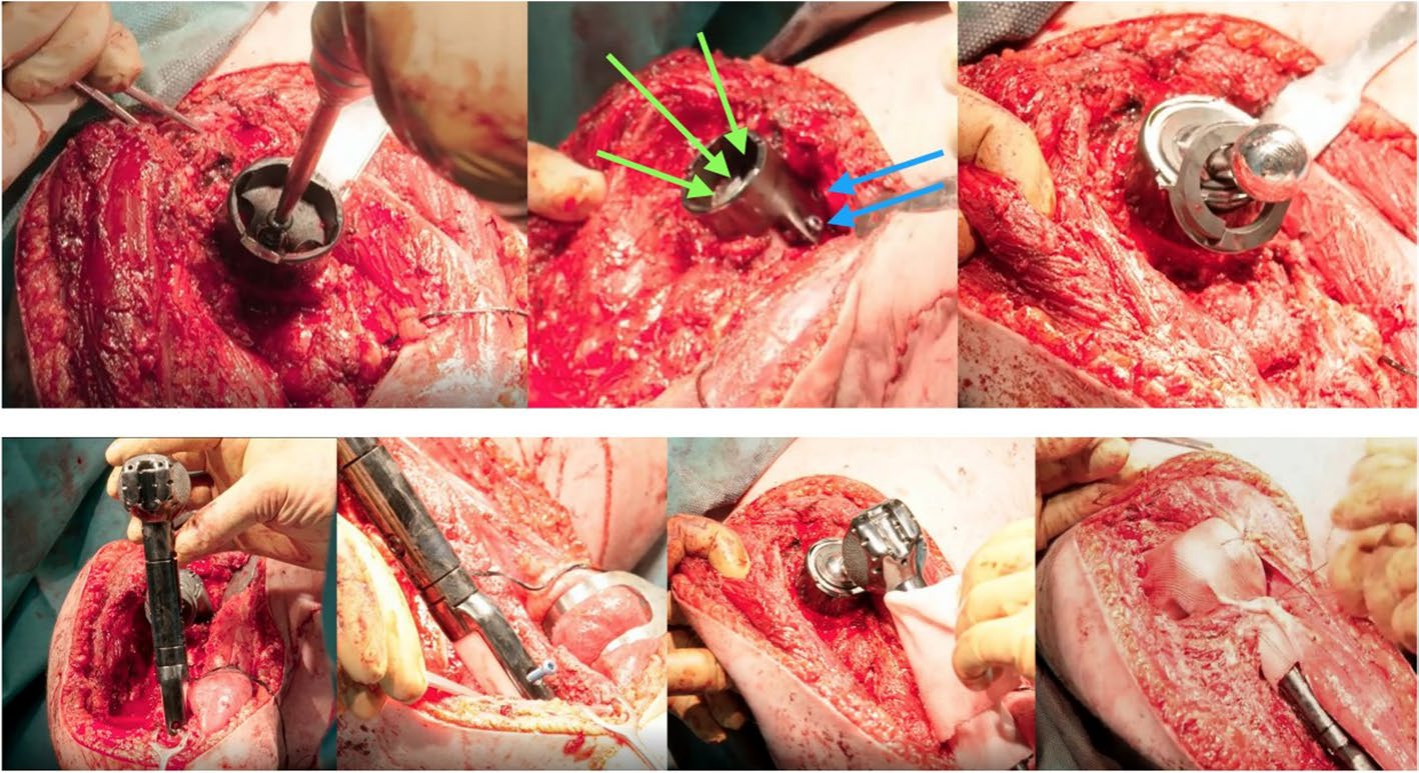
Surgical steps for implant fixation. Superior images demonstrating the fixation of the glenoid component with two sets of perpendicular screws (arrows) and the placement of the proximal end of the dumbbell connector. Inferior images showing the humeral component fixation, final connection of the dumbbell component, and closure with the use of a synthetic mesh. Green arrow, 3 planar screws through the glenoid component. Blue arrows, 2 perpendicular screws for enhanced fixation of the glenoid component to the native scapula

For all patients, CT scan images were obtained to design the custom-made implants. Initially, a 3D plastic dummy was printed and assessed for feasibility by the surgical team (Fig. 3), and once the model was approved, the actual implant was additively manufactured in modular components in a period of 6 weeks (Fig. 4). All patients were placed in a beach-chair position, and a delto-pectoral approach was performed with variable extension depending on the tumor expansion. Once the tumor was resected, the implant was trialed and secured in place. Hybrid fixation with uncemented glenoid component was used in all three cases. First, the glenoid/scapular component is fixed to the remaining scapula, and the proximal end of the dumbbell component is locked. The humeral component is then positioned and fixed into the medullary canal with cement and two locking screws. Finally, the distal end of the dumbbell is locked to the humeral component. A synthetic mesh was used to enhance soft tissue attachments to the implant (Fig. 4). Patients were seen to follow up at 2 and 6 weeks for wound assessment. Physical therapy was allowed after 6 weeks starting with pendulum exercises. Patients were assessed for oncologic outcome and for function using the Musculoskeletal Tumor Society (MSTS) scoring system. Additional outcomes of interest included surgical time, blood loss, pain, local recurrence, and complications.

## Results

**Fig. 5:**
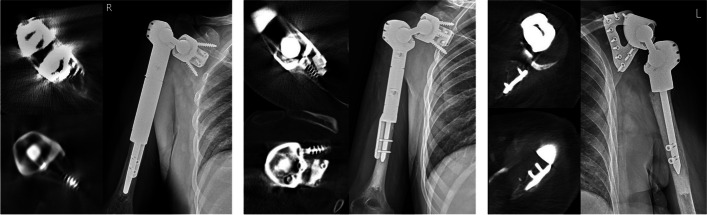
Final follow-up CT scans and radiographs. Imaging studies from all three patients at the time of last follow-up clinical visit (both CT and X-ray) demonstrating no signs of loosening of the glenoid component

**Fig. 6:**
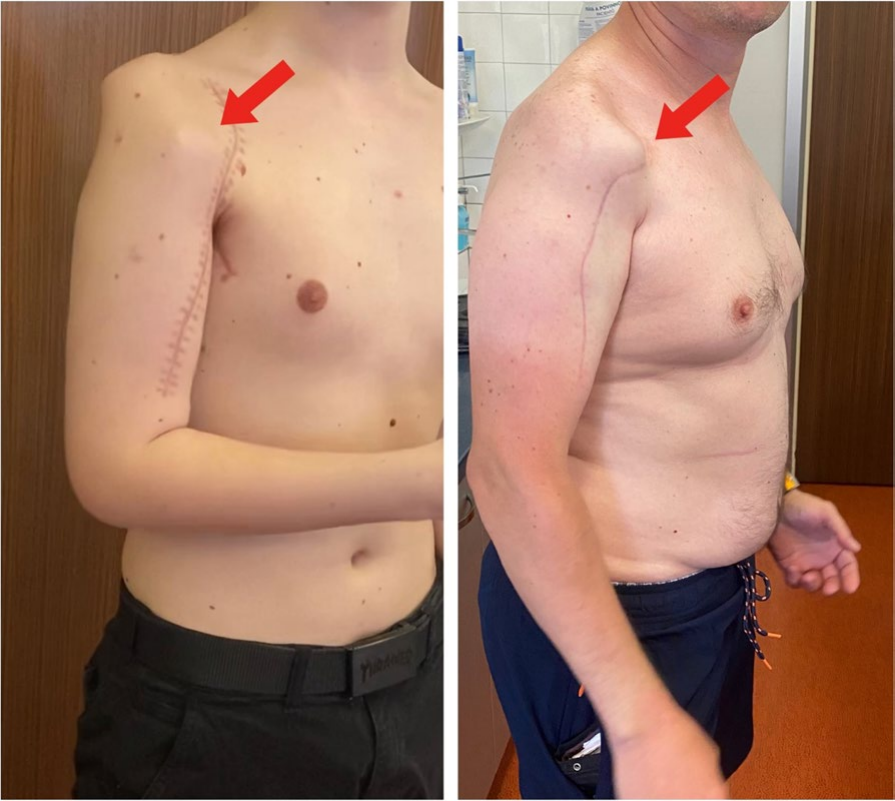
Patient in sagittal profile. Picture demonstrating mildly protruding proximal aspect of the implant of patient 2 (on the left) and patient 1 (on the right)

**Fig. 7:**
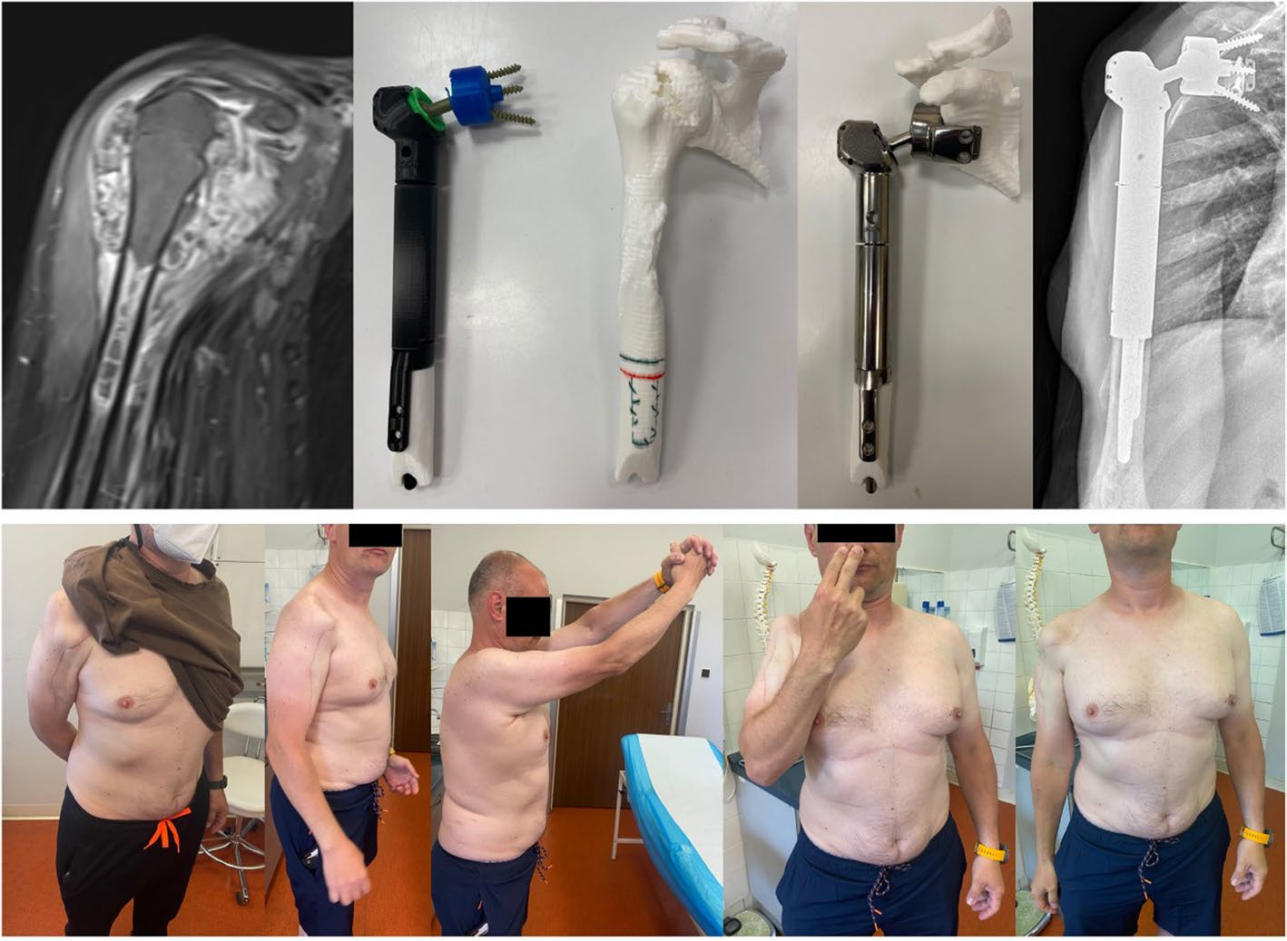
Case example patient 1. Pictures demonstrating the initial tumor presentation on MRI. Dummy and final implant printed. Postoperative radiograph showing good implant positioning and fixation. Clinical images of the patient’s postsurgical right arm function

The average surgical time was 175 min (range 150 to 190 min), and the average estimated blood loss was 516 mL (range 150 to 1000 mL). At the time of data collection, all patients were pain-free and had no local recurrences. No acute complications occurred. All three patients had good function and averaged at 21/30 points on MSTS score (Table 1). All patients had full elbow, wrist, and hand function that allowed them to carry out everyday activities. The dumbbell prosthesis remained stable, and no dislocations were reported. CT scans and radiographs were obtained at the last follow-up of each patient where no signs of loosening were observed (Fig. 5). On physical examination, all patients had a stable shoulder joint throughout passive range of motion. Passive abduction was up to 95° at which point acromion-implant impingement was noted. All three patients had a similar passive flexion of 120° degrees, passive internal rotation up to T12, and 40° of passive external rotation, which was dictated by the implant design. Two patients brought out the altered shoulder figure with a mildly protruding implant but was well tolerated and accepted (Figs. 6 and 7).

## Discussion

Reconstructing the proximal humerus after tumor resection poses a technical challenge for oncology orthopedists, especially in cases of extra-articular resection and for young patients with a good oncologic prognosis. Several alternatives are currently available, all with specific indications, advantages, and disadvantages [25, 26]. Some advocate the use of biologic options for younger patients, with more healing potential, whilst indicating endoprosthetic reconstruction for older patients with possibly lower functional requirements [27]. Liu et al. compared endoprosthesis to recycled autografts, finding high rates of revision for both reconstruction methods, however, with no significant differences among groups, reinforcing the need for improved alternatives for oncologic patients [28]. The stage of the tumor as well as its volume and type of resection was shown to be correlated to patients’ survival in one study also showing high rate of complications for extra-articular tumor resections [11]. Even though endoprosthetic replacements seem to be a good option for patients requiring an intra-articular resection where the deltoid muscle and axillary nerve can be preserved, the results are not translatable to cases of more extensive extra-articular resections [16, 29]. Furthermore, extra-articular resections were associated with high rates of local recurrences, a fact that possibly reflects the complexity of such tumor excision surgeries [30].

Among the biologic reconstructive alternatives, allograft is a popular choice, particularly in regions with limited resources. The accessibility to a bone bank allows for readily available allografts that when matched by CT scan 3D images can perfectly fit the patient enhancing healing rates [31]. Nonetheless, this alternative is not without complications, and the most frequent ones in the proximal humeral location seem to be allograft and subchondral fractures as well as reabsorption of the epiphysis [32]. Also obtaining an allograft with tendon attachments is usually available only after request, and its availability and quality can be less reliable than a metal implant. Recycled autograft is a niche indication with a similar complication profile to a frozen massive allograft. These techniques apply more to patients where the native joint can be preserved [33].

The allograft-prosthesis composite has long been proposed as a solution to proximal humeral resections. This combination of an allograft to a proximal humerus replacement adds the advantages of both components, as well as its disadvantages. Patients with this type of reconstruction can suffer prosthetic loosening as well as the non-union of the allograft. Moreover, as with other reconstruction alternatives, patients have a better functional outcome when an intra-articular resection is possible and when the abductor mechanism can be preserved [34].

Endoprosthetic replacements have evolved throughout the years to include better materials with improved implant survival [35]. However, patients continue to have a high risk of revision, especially during the first year after surgery and for patients with extra-articular tumor resections [36]. For the anatomic shoulder designed prostheses, the main complication is the high rate of dislocations, to the point that some authors suggest using it as spacer rather than actual shoulder arthroplasty solution [7, 27, 37]. To reduce instability of the shoulder prosthesis, use of a synthetic mesh was advocated to enhance soft tissues attachment [38]. Nonetheless, patient function continued to be limited, resulting in decreased range of motion (ROM) and no ability to perform overhead activities [9, 39]. To improve the overhead ROM, reverse shoulder arthroplasty has shown improved function when indicated due to rotator cuff pathology and osteoarthritis [40, 41]. Unfortunately, in tumor patients, the common complication continues to be instability [42, 43]. Moreover, reverse shoulder implants require a functional deltoid and axillary nerve to deliver its functional benefits, a scenario often not possible in the settings of extra-articular resections. Lastly, there is the alternative of a constrained reverse shoulder arthroplasty, promising to reduce instability rates. Unfortunately, recent studies have shown that this alternative has a higher-than-expected revision rate, most likely due to loosening of the implant [8].

The innovative implant presented here has potentially several advantages for the cases of patients requiring a Malawer type V resection [44] with resection of the axillary nerve and rotator cuff tendons. Given these patients will have an extremely limited active range of motion, the implant would act primarily as a fulcrum to more effectively position the hand in space in patients that otherwise would have a flail limb, unstable shoulder, or be at very high risk of aseptic loosening of the standard constrained implant. The implant has a diamond-like carbon surface which is biocompatible and has antibacterial properties potentially reducing the risk of postoperative infections, a feared complication in megaprostheses [45–47]. The polyetheretherketone (PEEK) inlay increases the durability of a constrained joint and shows less wear when compared to more frequently used polyethylene [48, 49]. The design of two ball and socket joints in a serial fashion is frequently used for engineering purposes where restrained multi-axis motion between two components is a necessity [50, 51]. According to our biomechanical analysis, using the new design significantly reduces the load transfer onto the glenoid component, hence decreasing the likelihood of the undesirable aseptic loosening [51, 52] whilst still providing a stable shoulder.

When compared to other surgical options, the constrained design eliminates the need for soft tissue balancing such as reconstructing the stumps of rotator cuff to the allograft in allo-prosthetic reconstructions or balancing the tension between components in reverse prosthesis. The fact that the humeral component is modular in our design facilitates planning, and unlike with allograft, there is less emphasis on the accuracy, fit, and finish of the osteotomy sites. There is virtually no learning curve with regard to the implant. The ease of use is reflected in the surgical times as well as average blood loss, which are at the lower range of what would be expected for these demanding surgeries.

The intent of the implant was to serve as a firm fulcrum to allow these patients with anticipated postoperative highly limited shoulder function, to have structural support that would enable the full utilization of the remaining upper extremity joints. The patients who received the innovative implant were able to carry weight through their elbows as well as to position their hands in the space to fulfill their daily activities successfully. All three patients at last follow-up had no imaging signs of implant loosening, were pain-free, had a high MSTS score, and were pleased with the results. The two patients who reported mild protrusion of the implant from the sagittal perspective reported this complaint as merely cosmetic and not interfering with everyday activities or life quality. Potential strategies to prevent this complication would be to perform local muscle transfers anterior to the implant (e.g., latissimus dorsi), when those remain after the extensive resection; however, this may not be an option for all patients, anterior fat grafting, or even a larger resection of the scapula to medialize the implant, the latter with a risk of further hindering the anchoring. Even though this study could be criticized for its limited number of subjects, the intent was not to provide a large case series but rather to serve as an initial proof of concept study for this novel implant. More patients continue to be added to our sample, and we continue to observe patients over time. Longer follow-up times and expanded sample results will be added to the literature when available. This will allow for a formal comparison among current different reconstruction alternatives in terms of implant survivorship and potentially different failure mechanisms of this novel implant.

## Conclusion

Our study demonstrates that this new implant is a promising alternative for reconstruction in the setting extensive extra-articular resections of the shoulder. The demonstrated decreased load transfer in between the components makes this an appealing option for young age patients with potentially good oncologic prognosis, where aseptic loosening of the glenoid component hinders the use of constrained implant. The dumbbell prosthesis is easy to use and yields good and reproducible functional results.

## Data Availability

Further case information is available from the corresponding author on reasonable request.
